# 3D genomic organization in cancers

**DOI:** 10.15302/J-QB-022-0317

**Published:** 2023-06-01

**Authors:** Junting Wang, Huan Tao, Hao Li, Xiaochen Bo, Hebing Chen

**Affiliations:** ^1^ Institute of Health Service and Transfusion Medicine Beijing 100850 China

**Keywords:** the three‐dimensional (3D) genome, chromatin compartment, topologically associated domain (TAD), loop, cancer

## Abstract

**Background:**

The hierarchical three‐dimensional (3D) architectures of chromatin play an important role in fundamental biological processes, such as cell differentiation, cellular senescence, and transcriptional regulation. Aberrant chromatin 3D structural alterations often present in human diseases and even cancers, but their underlying mechanisms remain unclear.

**Results:**

3D chromatin structures (chromatin compartment A/B, topologically associated domains, and enhancer‐promoter interactions) play key roles in cancer development, metastasis, and drug resistance. Bioinformatics techniques based on machine learning and deep learning have shown great potential in the study of 3D cancer genome.

**Conclusion:**

Current advances in the study of the 3D cancer genome have expanded our understanding of the mechanisms underlying tumorigenesis and development. It will provide new insights into precise diagnosis and personalized treatment for cancers.

## Abstract 2

**Author summary:** This review focuses on the role of 3D chromatin structures in cancer development. We also summarized common bioinformatics techniques, especially machine learning and deep learning methods for studying 3D cancer genome, and introduced their limitations.

## INTRODUCTION

**Figure 1:**
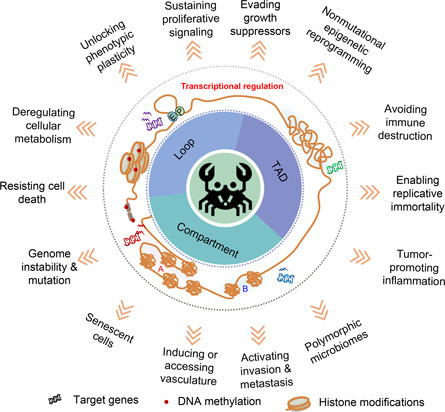
**The map of 3D genome in cancer.** 3D genomic structural changes in cancer promote the acquisition of oncogenic phenotypes by affecting the transcription of target genes.

Cancer is a malignant disease with high mortality rate which endanger human health worldwide. In a broad sense, cancer usually refers to all kinds of malignant tumors which are the product of continuous malignant proliferation of cancer cells [1]. Hallmarks of cancer are considered to be a set of capabilities critical to cancer formation in which human cells shift from normal state into tumor growth state. These hallmarks include self‐sufficiency in growth signals, insensitivity to anti‐growth signals, evading apoptosis, limitless replicative potential, and tissue invasion and metastasis, etc. [2, 3, 4]. Recently, the important role of three‐dimensional (3D) genome organization in cancer development was highlighted [5,6]. 3D genome variation in cancer cells can drive the acquisition of cancer hallmarks by affecting gene expression ( Fig.1).

**Figure 2:**
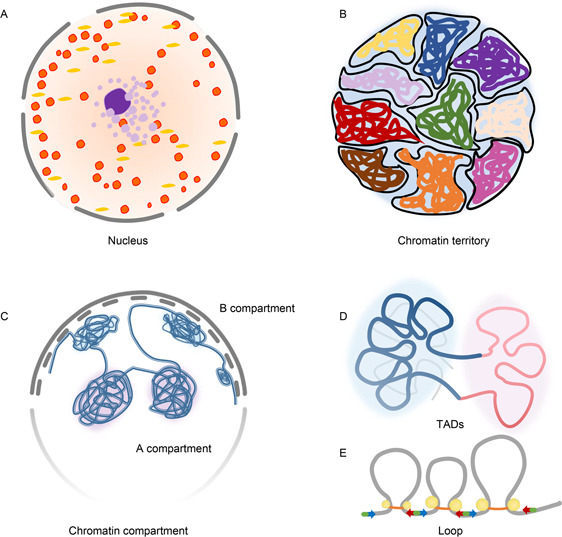
**Three‐dimensional folding of chromatin.** The hierarchical 3D chromatin stuctures, including (A) nucleus, (B) chromatin territory, (C) compartment, (D) TAD, and (E) loop. The yellow dots represent the transcription factor CTCF, red and blue arrows indicate the binding direction of CTCF, respectively. The orange ring represents the cohesin complex.

Technological innovations, such as high‐throughput chromosome conformation capture (Hi‐C) [7], chromatin interaction analysis by paired‐end tag sequencing (CHIA‐PET) [8] and protein‐centric chromatin conformation assay (HiChIP) [9], have revealed the hierarchical structure of 3D genome organization ( Fig.2) [10, 11, 12]. Each chromosome in the nucleus occupies a relatively independent region, chromosome territory ( Fig.2). It is the first step for us to understand the spatial structure of chromatin [13]. Then, the genome is organized into compartment A or B, which refers to active and inactive chromatin states, respectively ( Fig.2) [14]. Transitions from compartment A to B are always associated with down‐regulation of gene expression [15]. The large‐scale A/B compartments are segregated into megabase‐sized topologically associated domains (TADs) and chromatin loops that typically occur within TADs ( Fig.2, E) [16]. TAD boundaries are demarcated by the CCCTC‐binding factor (CTCF)/cohesin complex [17,18]. Genes within the same TAD tend to be co‐expressed during cell differentiation [19]. When TAD strays to the edge of the nucleus, the expression of its’ internal genes is suppressed, when it enters the central region of the nucleus, the gene expression becomes active [20]. Though TADs remain largely stable across distinct cell types and species [21], the epigenetic states and cohesin‐associated interaction loops within TADs show cellular heterogeneity [22,23]. Loop is a fundamental spatial regulatory structure that generally occurs between enhancers and promoters within TADs, which is necessary for gene expression [23]. Existing study has demonstrated the important regulatory role of 3D chromatin structure in gene expression [24]. It is reported that perturbations of 3D genome organization caused by the loss of H3K9 methylation activity will affect the gene expression patterns [25]. The interaction network between super enhancers (SE) and super silencers, as well as key transcription factors (TFs) also can regulate gene expression [26]. The 3D structure of chromatin provides the appropriate structural basis for TF‐ and epigenome‐mediated transcriptional regulation, and precisely regulates the expression of target genes through chromatin loop structure [27].

Cancer development is often accompanied by dramatic changes in the three‐dimensional structure of chromosome [28]. Aberrations in 3D genome are one of the major drivers promoting oncogenic transformation of normal cells by disturbing gene expression [29]. Chromatin topological changes alter the regulatory environment of target genes, which finally affect gene expression and drive cancer development [30]. Simultaneously, gene mutations can lead to aberrant phenotypes by affecting spatial genome folding [31]. It is reported that human papillomavirus (HPV) integrates in the cervical cancer genome and promotes local alterations in the expression of genes associated with tumor viability through chromatin reorganization [29,32]. Given the important regulatory role of 3D chromatin structure in gene expression, studying its role in cancer will help us understand the molecular mechanism of cancer occurrence and development.

The rapid development on 3D genome research benefits from the application of artificial intelligence (AI) [33,34]. Machine learning and deep learning‐based computational methods for the identification of 3D chromatin structures provide an excellent opportunity to explore 3D genome changes in cancer cells [35, 36, 37, 38, 39]. Moreover, computational tools have been developed to enhance the sequencing depth of Hi‐C data which make up the experimental limitations to some extent [40]. AI improves our understanding of hidden patterns in large and complex genomics data sets from cancer patients.

Growing number of studies have revealed the role of 3D genome in cancer development. We summarized hierarchical chromatin structure changes in cancer and described how these changes drive tumorigenesis and development. It will facilitate our understanding of mechanism underlying cancer development from the perspective of 3D genome, and provide new insights into precise diagnosis and personalized treatment for cancer. We also summarized AI models for identifying 3D genome and Hi‐C data enhancement. Applying these computational methods, we can further interpret the unique role of 3D genome in cancer development.

## COMPARTMENT SWITCH IN HUMAN CANCERS

**Figure 3:**
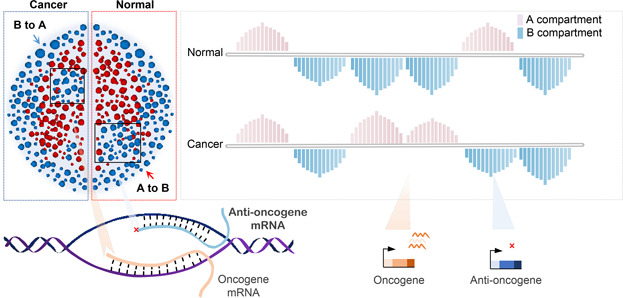
**Switching of chromatin compartment A or B is usually accompanied by activation or repression of genes.** Pink: A compartment, Blue: B compartment. The height of the bar represents the strength of the signal value. Shades of different colors correspond to different gene transcription situations.

In 2009, Aiden *et al*. investigated the 3D structure of human lymphoblastoid cells using Hi‐C and introduced the concept of the A/B compartment ( Fig.3) [14]. Subsequently, Barutcu *et al*. revealed that the A/B compartment switch between normal cells and breast cancer cells is associated with expression changes of corresponding genes [41]. It is reported that 20% of compartments undergo switch in myeloma which directly affects the expression of genes within corresponding compartments [15]. Besides, intermediate compartment (I compartment) was observed in colorectal adenocarcinoma which can be transformed into cell‐type‐specific A or B compartments [42]. Reportedly, the I compartment converge more closely to the A compartment in normal cells, while they are generally hypomethylating and converge more closely to the B compartment in cancer cells. It suggests that extensive compartmental remodeling is associated with tumor‐suppressive effects in which genes corresponding to stemness and invasion are inhibited, and anti‐tumor immunity genes are induced [42]. Blasi *et al*. found a highly dynamic I compartment which enriches H3K27me3 in poised promoters and polycomb‐repressive chromatin states in different subtypes of chronic lymphocytic leukemia and sarcoid lymphoma [43]. Disease‐specific changes in 3D genome often involve in extensive transcriptional activation of genes, including oncogenes associated with lymphomagenesis [43].

## TAD ALTERATIONS IN HUMAN CANCERS

**Figure 4:**
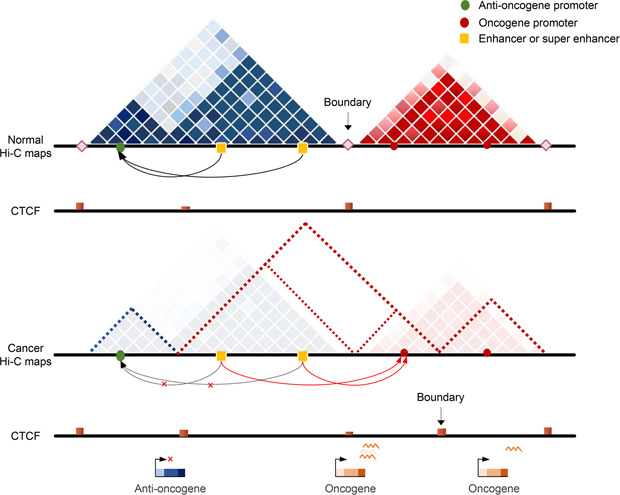
**Reorganization of TAD or subTAD in cancer leads to enhancer hijacking.** Hi‐C maps show the reorganization of TAD and subTAD in normal and cancer tissues. Green dots and red dots represent anti‐oncogene and oncogene promoters, respectively. The yellow squares represent enhancer or super‐enhancer. Diamond squares represent TAD boundaries. The height of the red bar indicates the CTCF signal value. Black arrows represent interactions existing in normal tissues and red arrows represent newly established interactions in cancer.

New technologies drive sequencing costs down even further, and increasing Hi‐C datasets emerge, which allows us to observe chromatin structure at a finer scale [21]. TADs organize the genome into ~Mb genomic regions separated by boundaries enriched with CTCF proteins, which have relatively frequent interactions with each other ( Fig.4). Its formation is largely driven by chromatin compartmentalization and loop extrusion [44]. Typically, TADs are isolated from each other by insulators, which contain one or several genes as well as their enhancers to form independent regulatory units [45]. Differences in 3D chromatin structure can affect the integrity of TADs and enhancer‐promoter interactions, which in turn affect gene expression and lead to human disease. However, how TADs are involved in cancer pathogenesis remains largely unknown.

In isocitrate dehydrogenase (IDH) mutated gliomas, mutated IDH leads to an abnormal increase in methylation at some TAD boundaries, which results in reduced binding of methylation‐sensitive CTCF to DNA [46]. It disrupts the formation of TAD boundary and further leads to a new remote regulatory interaction between enhancer and a glioma oncogene, platelet‐derived growth factor receptor A (PDGFRA) [46]. It increases the expression of PDGFRA and promotes the development of glioma [46]. Significant differences in TAD structure have also been reported between normal T and T‐ALL cells [47]. V‐Myc avian myelocytomatosis viral oncogene homolog (MYC) is one of the major oncogenes activating NOTCH1 signaling pathway which is usually up‐regulated in T cell acute lymphoblastic leukemia (T‐ALL) [48,49]. Kloetgen *et al*. found that repeated TAD fusions and increased intra‐TAD interactions in the *MYC* locus exist in all T‐ALL samples [47]. In addition, they highlighted that small molecule inhibitors can treat leukemia by altering specific regulatory patterns targeting 3D genome [47]. Accumulation of genomic mutations is common in human cancer development [50]. It is reported that the distribution of somatic mutations in cancer genome corresponds to spatial chromatin organization and the somatic mutation frequency is more pronounced at TAD boundaries [51]. Besides, Du *et al*. revealed the complex interaction between structural variation (SV) and chromatin 3D structure in pancreatic ductal adenocarcinoma (PDAC) [52]. They proposed that the TAD structure could confine SV within TADs to maintain genome stability [52].

With the development of computational methods for TAD prediction, researchers discovered smaller subTADs within conventional TADs [53]. LIM domain‐binding protein 1 (LDB1) maintains the subTAD structure which locates around 75 kb near Purine Rich Box‐1 (PU.1) during myeloid granulocyte differentiation [54]. Deletion of LDB1 destabilizes PU.1 localized subTAD, leading to the development of acute myeloid leukemia (AML) [54]. Increasing evidence suggests that oncogene‐induced senescence (OIS) is an important tumor suppressor mechanism [55]. SubTAD reorganization is considered as the initiating factor driving cells out of the oncogene‐induced senescence and acquiring invasive traits [56].

## ABNORMAL LOOPS IN HUMAN CANCERS

**Figure 5:**
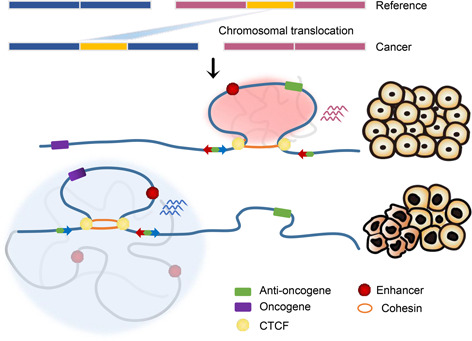
**Abnormal E‐P loop activate oncogene expression in cancer.** Structural variants (e.g., chromosomal translocation) in chromatin are able to regulate the formation of enhancer‐promoter (E‐P) loops, thus affecting gene expression and driving cancer development. The dark blue and magenta bars represent different chromosomes, the yellow bars represent chromosome translocation regions. Green bar and purple bar represent anti‐oncogene promoters and oncogene promoters, respectively. The red dots represent enhancers. The yellow dots represent the transcription factor CTCF, red and blue arrows indicate the binding direction of CTCF.

Chromatin loops allow distal chromatin regulatory elements, such as enhancers and promoters, to interact to regulate gene expression [23]. CTCF and cohesion complexes are key factors for chromatin loop formation [7]. Abnormalities of chromatin loops are also closely related to cancers in which enhancer hijacking is the most important mechanism ( Fig.5) [57]. The genetic mutations can cause the rearrangement of regulatory elements that promoters cannot interact with corresponding enhancers and finally result in gene misexpression [58]. It is reported that the chromatin structure surrounding the androgen receptor (AR) locus is altered to form many cancer‐specific enhancer‐promoter (E‐P) loops in prostate cancer cells [59]. Recent studies have shown that a large number of non‐coding region mutations and genomic rearrangements in tumors, which result in abnormal E‐P interactions [60,61]. In T‐ALL, Yang *et al*. found that chromatin translocations could mediate the formation of “neo‐Loop” and “neo‐TAD”, which activates the expression of the key gene *HOXA13* through enhancer hijacking [57]. Besides, the chimeric oncoprotein NUP98‐HOXA9 induces the formation of abnormal loops on oncogenes and leads to stronger activation of oncogenes through phase‐separated structures in AML [62]. Chu *et al*. revealed a 3D genomic pattern of STAG2 regulation in melanoma [63]. The enhancement of the H3K27ac‐associated DNA loops increases the expression of IRF9 and PD‐L1 which facilitates the immune evasion of STAG2‐mutant cancer [63]. It illustrates how cancer cells achieve immune escape and self‐protection through adaptive immune resistance from the perspective of 3D genome [63].

## COMPUTATIONAL TOOLS FOR IDENTIFYING CANCER 3D GENOME

**Figure 6:**
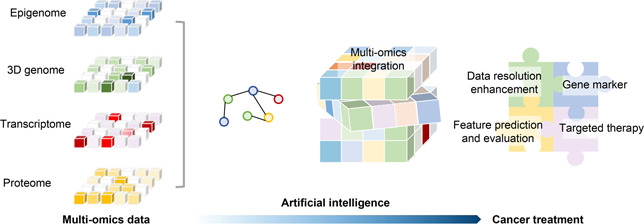
**Multi‐omics data integration based on AI promotes cancer research.** The integration of cancer multi‐omics data through AI facilitates deeper understanding of the cancer genome and contribute to cancer treatment.

AI models based on machine learning and deep learning show prominent prediction capabilities in the field of 3D genome ( Fig.6) [35, 36, 37, 38, 39]. It not only can identify hierarchical 3D chromatin structures, including chromatin compartments, TADs, and loops, but also can improve the Hi‐C data with low‐resolution. The application of AI models greatly promotes the study on 3D cancer genome. Exploring the 3D cancer genome enables precise and efficient exploration of chromatin structure alterations in cancers, thereby refining the 3D genomic regulatory landscape of cancer.

### Identification of chromatin compartments

For a long time, the mainstream compartment detection method has been developed based on principal component analysis (PCA) dimensionality reduction, using the first dimension to distinguish A/B compartments, such as Juice‐Box [64], HOMER [65] and Fan‐C [66]. However, it is difficult to dissect the biological meaning of the first dimension and the calculating speed is relatively slow. To address these issues, Zheng *et al*. developed CscoreTool, a statistical model to infer A/B compartments from Hi‐C data, which achieved a 30× increasing in speed and memory‐efficient [67]. Besides, SNIPER [68] and Calder [69] were developed for the identification of subcompartments using moderately covered Hi‐C data. Pentad can reliably detect the redistribution of contact frequency in the chromatin compartments and assess alterations in the compartment strength [70].

### Identification of TADs

In 2018, Chen *et al*. proposed HiCDB, a computational method for detecting TAD boundaries using local relative insulation metric and multi‐scale aggregation approach [71]. Then, advances in computational technology have led to the realization that TADs are not disjoint structural elements, but rather hierarchically organized domains [72]. OnTAD was developed for identifying hierarchical TAD structures [73]. OnTAD can identify candidate TAD boundaries by scanning genomes with a series of sliding windows of different window sizes [73]. Then, the candidate boundaries are assembled into an optimized hierarchical TAD structure using a recursive dynamic programming algorithm based on a scoring function [73]. Using OnTAD, Du *et al*. found that TAD nesting level could distinguish primary colorectal tumor tissue, normal colon tissue, colorectal cancer cell line and normal cell line [74]. Besides, changes in TAD hierarchy will affect the prognosis of colorectal cancer patients by altering gene expression [74]. In addition, Guo *et al*. constructed the TAD boundray alternation‐related gene identification in tumors (TARGET) based on chromatin spatial structure and transcriptome data [75]. TARGET can identify TAD boundaries that are specifically altered in tumors and predict aberrantly expressed candidate genes in tumors that are regulated by aberrant alterations in TAD boundaries [75]. The spatial density of the open chromatin (SDOC) metric was proposed as a quantitative measure of intra‐TAD chromatin state and structure to sensitively reflect epigenetic properties and gene transcriptional activity in TADs [76]. SDOC can facilitate the identification of the alterations in the internal densities of TADs whose TAD boundaries remain unchanged during tumor evolution to underlie transcriptional regulatory mechanisms of oncogenic transformation [76].

### Identification of chromatin loops

To study the regulatory role of chromatin loops in the development of cancer, Wang *et al*. proposed NeoLoopFinder, to identify enhancer hijacking on cancer genomes based on Hi‐C data [77]. It can identify SV‐mediated loop by removing the data bias caused by copy number variation, SV heterozygosity, and heterogeneity [77]. NeoLoopFinder has been widely used in the study of muscle‐invasive bladder cancer [78] and diffuse intrinsic pontine glioma [79]. Loops between subtype‐specific promoter and enhancer can regulate key oncogenes and drive oncogenic progress by increasing the contact of linear distant between regulatory elements and target genes in muscle‐invasive bladder cancer [78]. Wang *et al*. also found that abnormal chromatin loops are associated with diffuse intrinsic pontine glioma (DIPG), which has the highest mortality rate among pediatric solid tumors [79]. The remodeling of the E‐P loop in DIPG cells can be inhibited by small‐molecule inhibitors or degraders [79].

Besides, Cameron *et al*. presented the Hi‐C interaction frequency inference (HIFI) algorithm, which can accurately estimate restriction‐fragment resolution Hi‐C matrices by exploiting dependencies between adjacent fragments [80]. FitHiC1/2 implements a statistical confidence estimation method to detect loops [81,82]. 3DPredictor uses CTCF binding signaling and gene expression to quantitatively predict chromatin interactions [37]. The ensemble machine learning model‐LoopPredictor can be applied to predict enhancer‐mediated genome‐wide interactions which can isolate cell type–specific gene regulatory networks from three different cancer cell lines [38]. Recently, EPIXplorer has been developed to predict long distance E‐P interactions which facilitate us understand how genome‐wide association study (GWAS) variants affect the development of cancer [39].

### Hi‐C data enhancement

There are a lot of Hi‐C data, but their resolution is generally limited, which has become a challenge in the 3D genome research. Deep learning and machine learning has been increasingly applied in computational tools for Hi‐C data enhancement, which can help us better explore the 3D genome in cancer. HiCPlus is a deep convolutional neural network‐based method to improve the resolution of Hi‐C data [83]. It is a pioneer to apply deep learning in improving the resolution of Hi‐C data. Then, methods for enhancing Hi‐C data have emerged, such as HiCNN 1/2 [84,85], hicGAN [86], and DeepHiC [87] in which hicGAN, and DeepHiC are both built based on generative adversarial networks. It is worth mentioning that DeepHiC provides a user‐friendly webserver that can enhance low‐resolution data in just a few simple steps [87].

## CONCLUSIONS AND FUTURE PERSPECTIVES

The occurrence of cancer is a multi‐factor, multi‐stage, complex and progressive process. The gradual in‐depth analysis of 3D cancer genome has enabled us to have a more comprehensive understanding of cancer development. 3D chromatin structures, such as A/B compartments, TADs, and loops, are dynamically linked, and synergistically regulated, which plays important roles in regulating gene expression and cancer development. Changes in 3D chromatin structures can directly affect the aberrant transcription of corresponding genes, thereby promoting oncogenic transformation. Besides, the emergence of AI‐based computational tools for identifying 3D chromatin structures and improving Hi‐C data provides the possibility to explore the role of 3D genome in cancer development.

Cancer exhibits strong heterogeneity in which different tumors, cell lines, and even molecular subtypes have completely distinct regulatory patterns [88]. The specific regulatory loop can enhance the expression of oncogenes through abnormal E‐P interaction. Reorganization of TAD or subTAD destroys the local stability of 3D genome, so that the variation on the genome cannot be localized. Switching of the A/B compartment often causes widespread transcriptional dysregulation in cancer cells. Moreover, only 2% of the genomic regions can encode proteins, and about 98% of the genome regions belong to the non‐coding region which contains a large number of regulatory elements [60,89]. Exploration of the genomic features and chromatin regulatory landscape of non‐coding regions from the perspective of 3D genome will provide a comprehensive view in cancer development. With the development of 3D genome research, there will be increasing methods to find the regulatory effect of chromatin structure. The chromatin structure is likely to become a new cancer detection marker and therapeutic target [90]. 3D genome study will not only facilitate us to understand the mechanism underlying tumor development, metastasis, and drug resistance, but also contribute to identify the molecular targets for cancer diagnosis and drug discovery.

**Figure 7:**
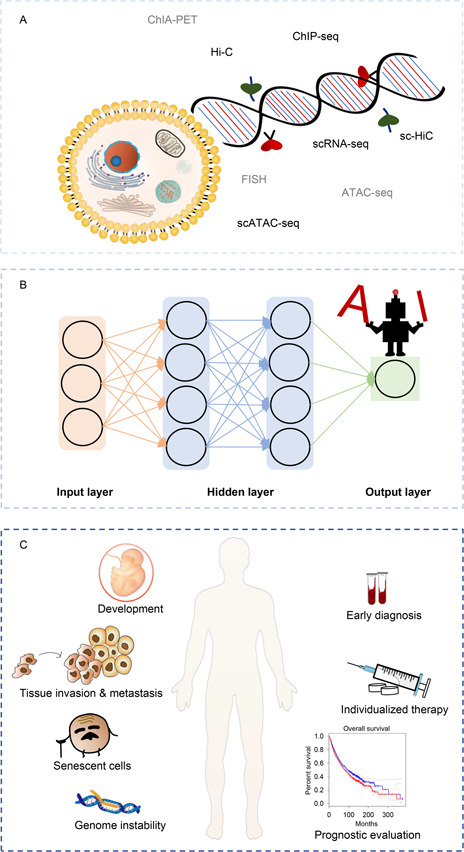
**New opportunities for cancer mechanism research and treatment.** (A) Multiomics data to study 3D genome. (B) AI‐based methods. (C) Application prospect of AI‐based methods in cancer research and treatment.

Despite the rapid development of 3D cancer genomics, there are still some limitations to overcome in the future. Firstly, traditional Hi‐C only reflect the average characteristics of a cell population, which cannot fully reveal the heterogeneous characteristics of tumors. In contrast, single‐cell Hi‐C is capable of resolving conformational models of individual chromosomes and elucidating chromosomal interactions and mechanisms regulating genomic function [91]. However, the high cost and cumbersome data processing process limit its wide application. Besides, it is difficult to explain the in‐depth mechanism underlying 3D genome variation in cancer. The continuous generation of cancer multiomics data and the development of AI have promoted the transformation of cancer research from low dimension to high dimension ( Fig.7, B). The application of AI promotes the process and integration of multiomics data which help us in understanding of the law of tumor occurrence and development. It will finally facilitate the diagnosis, treatment, and prognosis improvement of cancer ( Fig.7, C). Moreover, existing methods still lack interpretability [92]. The development of interpretable AI models can make models more transparent and logical to further facilitate the integration of massive sequencing data. AI with multidimensional regulatory information in oncology will provide a unique interface to reveal the black box of cancer precision therapy.

From the vast universe to the extremely tiny cells, human beings are constantly recognizing and transforming the world. The exploration of the human genome is still in the initial stage. We believe the regulatory landscape of cancer genomes will be more comprehensively described in the future. There will be a day that cancer is no longer an incurable disease.

## COMPLIANCE WITH ETHICS GUIDELINES

The authors Junting Wang, Huan Tao, Hao Li, Xiaochen Bo and Hebing Chen declare that they have no conflict of interest or financial conflicts to disclose.

This article is a review article and does not contain any studies with human or animal materials performed by any of the authors.
